# Alleged Malpractice in Orthopedic Surgery in The Netherlands: Lessons Learned from Medical Disciplinary Jurisprudence

**DOI:** 10.3390/healthcare11243111

**Published:** 2023-12-07

**Authors:** Netanja I. Harlianto, Zaneta N. Harlianto

**Affiliations:** 1 Department of Orthopedic Surgery, University Medical Center Utrecht and Utrecht University, 3508 GA Utrecht, The Netherlands; 2 Faculty of Medicine, Anton de Kom University, Paramaribo 9212, Suriname

**Keywords:** jurisprudence, malpractice, medical errors, medical liability, orthopedic surgery

## Abstract

Background: Orthopedic surgery is a specialty at risk for medical malpractice claims. We aimed to assess the frequency of alleged malpractice cases related to orthopedic surgery in the Netherlands from the last 15 years. Methods: We systematically searched the database of the Dutch Medical Disciplinary Court for verdicts related to orthopedic surgery between January 2009 and July 2023 and extracted case data and data on allegations and outcomes. Results: We identified 158 verdicts (mean of 10.5 per year), of which 151 (96%) were filed against specialists and 7 (4%) against residents. Cases were most frequently classified as incorrect treatment/diagnosis (*n* = 107, 67.7%). Cases were related to the subspecialties of knee (*n* = 34, 21.5%), hip (*n* = 31, 19.6%), ankle (*n* = 25, 15.8%), spine (*n* = 22, 13.9%), and shoulder (*n* = 19, 12.0%). A total of 32 cases (20.3%) were judged as partially founded and 9 (6%) as founded. The Dutch Medical Disciplinary Court imposed 28 warnings, 10 reprimands, and 3 temporary suspensions. A total of 68 appeals were submitted, of which 95% were rejected for filing patients. In three instances, unfounded verdicts were changed to two warnings and a reprimand. In four appeals by an orthopedic surgeon, a warning and reprimand were dismissed, and two reprimands were changed to warnings. Conclusions: The amount of malpractice cases against orthopedic surgeons in the Netherlands is relatively low. The cases in our study may improve our understanding of allegations against physicians and improve the quality of patient care.

## 1. Introduction

The field of orthopedic surgery is regarded as a field with increased risks for malpractice claims. In a USA-based study conducted by Jena et al. [1], orthopedic surgeons were twice as likely to be subject to a lawsuit compared to the average across all specialties (14% vs. 7.4%). In order to claim malpractice, the court usually has to establish that a defendant acted negligently. Negligence is defined as conduct that falls below the minimum degree of ordinary care imposed by law to protect patients against unreasonable risk of harm, without wrongful intent [2]. Reasons for filing malpractice suits may include wanting financial compensation, to hold a physician accountable for their actions, and to find explanations in the case of unwanted adverse events or complications [2]. These claims can have negative effects on the physician–patient relationship [3]. Moreover, in attempting to minimize malpractice risk, orthopedic surgeons more often practice “defensive medicine”. This includes ordering additional examinations and tests to primarily avoid malpractice cases [4,5]. It is estimated that, in the USA, the added cost for defensive medicine is USD 2 billion annually [6].

While previous studies have assessed malpractice risk in the USA [7] and across Europe [8,9,10,11], alleged malpractice claims in the field of orthopedic surgery in the Netherlands are still unreported. Malpractice jurisdiction in the Netherlands primarily aims to withhold and constantly improve the quality of care. It is not the goal of the system to financially compensate patients or to penalize physicians, which may be different compared to other countries in Europe and around the world [12]. Financial compensation following medical errors in the Netherlands is handled in civil court, by the hospital’s insurance company, or the conciliation board, which is separate from alleged malpractice claims [12].

Insight into alleged malpractice may improve our understanding of allegations against physicians and improve the quality of care for the orthopedic patient population.

Hence, the purpose of this study was to systematically evaluate alleged malpractice cases handled by the Dutch Medical Disciplinary Court in the field of orthopedic surgery and to describe the extent of allegations, outcomes, and disciplinary measures imposed in the last 15 years.

## 2. Materials and Methods

### 2.1. Database and Data Collection

The online database of the Dutch Medical Disciplinary Court (https://tuchtrecht.overheid.nl/, accessed on 4 August 2023) was systematically investigated for verdicts made available between January 2009 and July 2023 against physicians in the field of orthopedic surgery. Malpractice cases against physicians in the Netherlands can be filed by patients, their immediate relatives, and by the Health and Youth Care Inspectorate, a governmental institution overseeing public health in the Netherlands. Each individual case is handled by two legal experts and three medical experts (most often in the field of orthopedic surgery). After each verdict, data on each case are published online after one day [13]. These data are publicly available and anonymized. Therefore, for our study, institutional review board approval was not necessary. Information was extracted related to the year of verdict, orthopedic surgery subspecialty, the number of months between the filing of the allegation and the date of the verdict, whether the defendant was a specialist or resident, the extent of the allegations, the use of an attorney by the patient and the defendant, the verdict, disciplinary measure, and appeal. The Dutch Medical Disciplinary Court can impose various consequences if the allegations against physicians are (partially) founded. These include a warning, reprimand, monetary fine, suspension, and practice prohibition [14,15]. A detailed description is shown in Table 1.

### 2.2. Statistics

Data analysis was performed using R Statistical Software version 4.2.2. (R Foundation for Statistical Computing, Vienna, Austria). Categorical data were presented as frequencies (%), and numerical data as median with interquartile range (IQR). We used Spearman’s Rho to determine whether the frequency of the number of alleged cases has remained stable over time. Differences in characteristics were compared between unfounded and (partially) founded verdicts using chi-squared tests for categorical data and the Wilcoxon signed rank rest for non-normal continuous data. Statistical significance was set at a *p*-value less than 0.05.

## 3. Results

### 3.1. Case Characteristics

**Figure 1:**
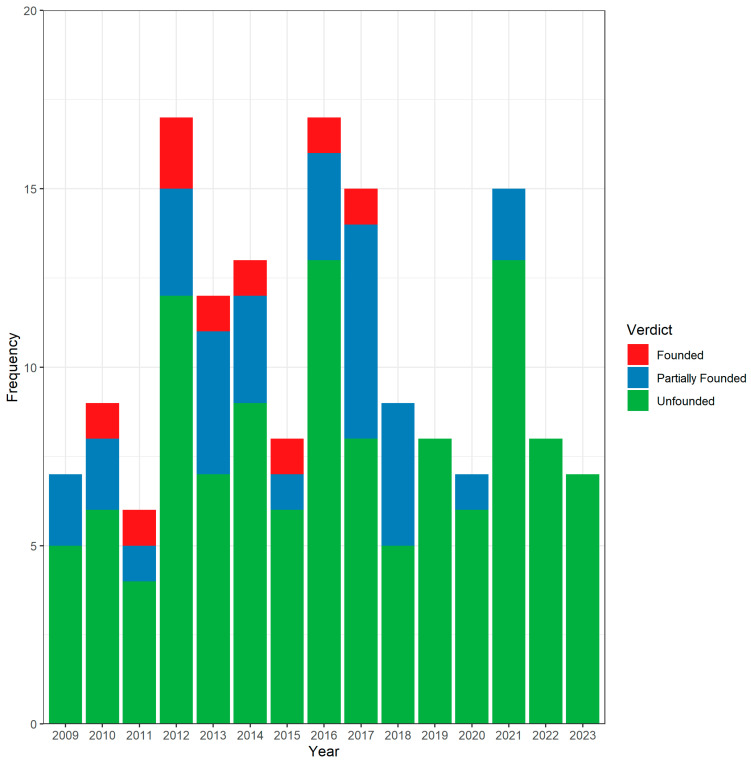
The number of verdicts between 2009 and 2023.

Between January 2009 and July 2023, 158 verdicts handled by the Dutch Medical Disciplinary Court related to orthopedic surgery were identified. The mean number of cases was 10.5 per year. No significant association was observed between the time and number of verdicts (Spearman’s Rho 0.05, *p* = 0.86). The amount of cases stratified by verdict over time are displayed in Figure 1. 

Seven cases (4%) were filed against orthopedic surgery residents, and 96% of cases were filed against orthopedic surgeons. Cases were classified as incorrect treatment/diagnosis (*n* = 107, 67.7%), no/insufficient care (*n* = 17, 10.8%), insufficient information (*n* = 10, 6.3%), incorrect statement/documentation (*n* = 8, 5.1%), other (*n* = 7, 4.4%), no/delayed referral (*n* = 4, 2.5%), improper treatment (*n* = 3, 1.9%), and violating patient–doctor confidentiality (*n* = 2, 1.3%). In general, this classification was not statistically different between founded and unfounded verdicts (*p*-value = 0.86). 

Stratified by subspecialty, most cases were related to the knee (*n* = 34, 21.5%) and hip (*n* = 31, 19.6%), followed by the ankle (*n* = 25, 15.8%), spine (*n* = 22, 13.9%), shoulder (*n* = 19, 12.0%), other (*n* = 13, 8.2%), hand (*n* = 7, 4.4%) oncology (*n* = 3, 2.5%), pediatrics (*n* = 2, 1.3%), and elbow (*n* = 2, 1.3%). For cases with a (partially) founded verdict, the most common involved subspecialty involved the hip (27%) and spine (27%), though this difference was not statistically significant compared to the unfounded group (*p* = 0.06).

A total of 73 patients (46.2%) had an attorney during the court hearing, a rate much higher for physicians, with 149 (94%) having an attorney. For cases with a (partially) founded verdict, the percentage of patients with an attorney was significantly higher compared to cases with an unfounded verdict (80% vs. 33.3%, *p*-value < 0.001). The percentage of physicians with an attorney was not significantly different between founded and unfounded cases (95% vs. 94%, *p* = 0.99).

The median time in months between the submission of the allegation and the verdict was 9.9 months (IQR: 7.6–12.5, range: 2.6–24.2 months). There were no statistically significant differences between founded and unfounded verdicts for the time between the submission of the allegation and the verdict (median 9.9 months; IQR: 7.5–12.8 vs. median 9.8 months; IQR: 7.6–12.2, *p*-value = 0.60). A summary of the characteristics between founded and unfounded verdicts is shown in Table 2. More detailed data on each case are provided in Supplementary Material Tables S1 and S2.

### 3.2. Outcomes

A total of 32 cases (20.3%) were judged as partially founded, 9 were judged as founded (6%), and the remaining 117 cases were judged as unfounded. The Dutch Medical Disciplinary Court imposed 28 warnings, 10 reprimands, and 3 suspensions for 1 month (*n* = 1), 6 months (*n* = 1), and 1 year (*n* = 1). A qualitative description of reasons for (partially) founded verdicts is provided in Table 3. A total of 68 appeals were submitted against the final verdicts, 9 appeals were filed by orthopedic surgeons, 56 by patients, and 3 by both physicians and patients. For filing patients, 95% of appeals were rejected. All patient appeals were rejected in unfounded cases, and granted for a subset of patients in cases with a (partially) founded verdict. In three instances, unfounded verdicts were changed to two warnings and a reprimand. In four instances where an orthopedic surgeon filed an appeal, a warning was dismissed (*n* = 1), a reprimand was dismissed (*n* = 1), and a reprimand was changed to a warning (*n* = 2). The remainder of appeals were rejected.

## 4. Discussion

In the current study, we showed that, on average, 10.5 cases of alleged malpractice have been filed against orthopedic surgeons or residents per year in the past 15 years in the Netherlands. The number of cases did not significantly change in frequency over time. There are approximately 1100 cases of alleged malpractice filed against physicians in the Netherlands each year across all specialties [16]. As the total number of registered orthopedic surgeons in the Netherlands is around 900 [17] and the number of alleged cases against orthopedic surgeons or residents accounts for less than 1% of all malpractice cases, the number of yearly allegations can be considered relatively low. When filing a malpractice allegation, patients are required to pay a court fee of EUR 50, which has to be paid again if an appeal is submitted against the verdict [18]. Ninety-four percent of physicians had an attorney, a proportion which was much lower for patients. Orthopedic surgeons may have a lawyer assist them during the hearing via insurance, the hospital, or on their own accord. If patients want legal assistance during the hearing, they have to hire a lawyer on their own accord. This requires additional costs, which may explain why this percentage was lower overall. In founded verdicts, a higher percentage of filing patients had an attorney when compared to filing patients with an unfounded verdict as a result. 

The majority of cases were classified as incorrect treatment/diagnosis (67.7%), followed by no/insufficient care (11%) and providing insufficient information (6%). Furthermore, most cases were filed against orthopedic surgeons, with the subspecialities of hip and knee surgery most commonly involved. In line with our study, hip- and knee-related pathologies were the most frequent litigated cases in orthopedic surgery in other cohorts from the USA, Italy, and France, which also included civil litigation [8,9,19]. Agout and colleagues analyzed 71 claims out of 51.582 surgical procedures over a 10-year period, which significantly increased over time [8]. A high percentage of malpractice claims were related to revision surgery, with the main reason for disputes being infections [8], complications frequently seen in lower limb arthroplasty [20]. Similar findings were seen when analyzing litigations related to knee and hip arthroplasty in France, with infections being the main cause of malpractice in 256 cases between 1990 and 2020. Infections as the cause of a malpractice claim in our analysis of the past 15 years was relatively rare.

Casali et al. [9] analyzed 635 claims in Italy between 2002 and 2023. They found that the malpractice was established in 52% of all cases and that the majority of claims were related to perioperative and operative care, and that, more specifically, sciatic nerve lesions were the most common cause of hip and knee involvement in malpractice. The reasons for founded verdicts in our cohort varied widely, ranging from preoperative factors such as incorrect diagnoses, not obtaining informed consent correctly, and not discussing the risks of surgery to operative factors such as incorrect surgical techniques or a wrong-sided operation. In contrast to our findings, a large retrospective analysis in the United Kingdom analyzing 8548 claims found that mismanagement was the main cause of litigation claims, followed by errors in diagnosis and perioperative factors [21]. Moreover, there was a significant increase in the number of claims over time [21]. These increases in malpractice claims across various countries in Europe are in contrast to the findings of our study. Likewise, malpractice claims have been reported for other specialties in the Netherlands, including radiology, gynecology, and emergency medicine [15,22,23]. Over time, the number of malpractice claims and cases has remained stable for radiology [15] and gynecology [22], whereas the number declined for emergency medicine [23]. These findings can be attributed to the unique design of the healthcare system in the Netherlands. The relatively low number of alleged malpractice claims may be a result of the way malpractice is structured in the Netherlands, allowing for the frequent assessment of errors and predominantly striving to improve the quality of care. In other European countries, efforts have been made with quality of care cycles to improve healthcare systems and improve patient care [24,25].

In our study, around 26% of malpractice cases against orthopedic surgeons were judged as (partially) founded. The majority of these orthopedic surgeons received warnings, and in fewer instances, reprimands, which indicated that they acted with culpable negligence. Three cases were more severely punished with temporary suspensions and the inability to practice. No orthopedic surgeons received monetary fines or permanent suspensions in the past 15 years. 

Previous work has evaluated the effect of alleged malpractice on physicians in the Netherlands [26,27]. The disciplinary process and risk of court measures can have significant psychological and professional impact on physicians. More specifically, respondents mentioned feelings of insecurity and misery and the fear of having new alleged malpractice cases filed against them. This is amplified by being seen as guilty before the final verdict is reached, the risk of having a permanent record of misconduct in the physician registry, online publication of the case in newspapers, and the extended timeframe before a verdict is reached by the court [26]. We identified that the median time before a verdict is reached is 9.9 months (IQR: 7.6–12.5), though this did not differ based on whether the outcome was unfounded or founded. 

Reprimands have greater negative effects on work and health than warnings. Interestingly, physicians who have been accused of alleged malpractice reported practicing “defensive medicine” more often by ordering additional examinations and complying with the demands of patients more frequently [27]. It would be of interest to explore the effect of alleged malpractice in the Netherlands on the orthopedic specialist and whether this has an impact on the practice of defensive medicine. Nevertheless, extensive analyses of medical negligence cases are important to identify certain risk patterns and to raise awareness of pitfalls in the treatment of orthopedic patients, minimizing the risk of future medical errors.

This is the first study to systematically investigate alleged malpractice, its characteristics, and its outcomes related to orthopedic surgery in the Netherlands. However, the limitations of our study warrant consideration. The results of our study are based on the Dutch healthcare system, which may not always be generalizable to other (European) countries. Secondly, while the Dutch Medical Disciplinary Court assesses whether an alleged malpractice case has culpable negligence, patients do not receive monetary compensation, which is usually settled in civil court. We were not able to include civil court verdicts in our study, as civil court cases are not always made available online, which would limit a systematic investigation. Previous studies have evaluated medical claims in the Netherlands for ankle surgery, for which the number of claims has remained stable between 2006 and 2016 with a median settlement of EUR 12,549 [28], which was EUR 5921 for patients filing after total hip replacement between 2002 and 2012 [29].

## 5. Conclusions

The number of alleged malpractice cases against orthopedic surgeons in the Netherlands is relatively low, with approximately 10.5 cases each year. Around one quarter of submitted appeals were (partially) founded. Knee and hip were the most frequent subspecialties involved. We highlighted the cases of alleged malpractice, which may improve our understanding of allegations against physicians and improve the quality of care for the orthopedic patient population.

## Figures and Tables

**Table 1:** Disciplinary measures imposed by the Dutch Medical Disciplinary Court.

I. Warning. This is a reproof for misconduct, but not culpable negligence.
II. Reprimand. This is a reproof for culpable negligence.
III. A monetary fine up to EUR 4.500.
IV. A (conditional) suspension for up to one year. This measure may also be imposed together with a fine.
V. Partial prohibition to practice.
VI. Complete prohibition to practice.

The list of measures is in order of severity. Warnings have no consequences and are never published. A reprimand is published for 5 years in the physician registry, and in special instances, in local newspapers, as decided by the Dutch Medical Disciplinary Court. Cases of a suspension or practice prohibition are published by default in the physician registry and local newspapers.

**Table 2:** Characteristics between unfounded and (partially) founded verdicts.

	Total Group (*n* = 158)	Unfounded Verdict (*n* = 114)	(Partially) Founded Verdict (*n* = 44)	*p*-Value
**Training level**				
Specialist	151 (96%)	108 (95%)	43 (98%)	0.78
Resident	7 (4%)	6 (5%)	1 (2%)	
**Subspecialty**				
Knee	34 (22%)	30 (16%)	4 (9%)	0.06
Hip	31 (20%)	19 (17%)	12 (27%)	
Ankle	25 (16%)	20 (18%)	5 (11%)	
Spine	22 (14%)	10 (9%)	12 (27%)	
Shoulder	18 (11%)	12 (11%)	6 (14%)	
Hand	7 (4%)	7 (6%)	0 (0%)	
Oncology	3 (2%)	3 (3%)	0 (0%)	
Elbow	2 (1%)	2 (2%)	0 (0%)	
Pediatric	2 (1%)	2 (2%)	0 (0%)	
Other	14 (9%)	9 (8%)	5 (11%)	
**Category**				0.86
Incorrect treatment/wrong diagnosis	107 (68%)	79 (69%)	28 (64%)	
Providing no or insufficient care	17 (11%)	13 (11%)	4 (9%)	
Providing insufficient information	10 (6%)	7 (6%)	3 (7%)	
Inaccurate statement or documentation	8 (5%)	5 (4%)	3 (7%)	
No or delayed referral	4 (3%)	4 (4%)	0 (0%)	
Improper treatment	3 (2%)	2 (2%)	1 (2%)	
Breach of confidentiality	2 (1%)	1 (1%)	1 (2%)	
Other	7 (4%)	3 (3%)	4 (9%)	
**Percentage of physicians with an attorney**	94%	94%	95%	0.99
**Percentage of patients with an attorney**	47.5%	33.3%	80%	<0.001
**Time between filing and verdict in months**	9.9 (IQR: 7.6–12.5)	9.8 (IQR: 7.6–12.2)	9.9 (IQR: 7.5–12.8)	0.60
**Number of appeals**	68 (43%)	48 (42%)	20 (45%)	
Rejected	61 (90%)	48 (100%)	13 (65%)	
Granted	7 (10%)	0 (0%)	7 (35%)	
**Appellant**				
Specialist	12 (17%)	0 (0%)	12 (52%)	
Patient	59 (83%)	48 (100%)	11 (48%)	

Abbreviations: IQR: interquartile range. Categorical data are shown as frequency (percentage), and numerical data as median (interquartile range). *p*-values are reported for differences between the unfounded and founded groups.

**Table 3:** Qualitative descriptions of founded verdict components.

Main Components of Founded Verdicts
Wrong diagnosis
Not performing additional imaging
Inadequate documentation in the electronic health record
Not adhering to orthopedic guidelines
Incorrect or insufficient acquisition of informed consent
Not discussing the risks of the operation
Incorrect operation indication
Incorrect time-out procedure, operating on the wrong side
Intraoperative errors
Incorrect surgical technique
Inadequate documentation of operation report
Inadequate postoperative care
Failure to refer to another specialist
Accessing the electronic health record and spreading information without consent

## Data Availability

Available upon reasonable request from the corresponding author.

## Supplementary Materials

The following supporting information can be downloaded at: https://www.mdpi.com/article/10.3390/healthcare11243111/s1, Table S1: A summary of all unfounded verdicts. Table S2: A summary of all founded verdicts.
