# Supplementary material for: Alleged Malpractice in Orthopedic Surgery in The Netherlands: Lessons Learned from Medical Disciplinary Jurisprudence
Source: Healthcare (Basel). 2023 Dec 7;11(24):3111. doi: 10.3390/healthcare11243111 (PMC10742425; doi:10.3390/healthcare11243111)
Supplement: Supplementary file 1 [file healthcare-11-03111-s001.zip › healthcare-2700613-supplementary.pdf]

Supplementary Table S1. A summary of all unfounded verdicts

Case	Year	Subspecialty	Defendant	Time between filing and verdict (months)	Allegation	Attorney patient	Attorney defendant	Verdict	Measure	Appeal	Appellant
1	2023	Spine	Orthopedic surgeon	6	Ignored symptoms and misapplied AMA-guides, incorrect history, stepped outside area of expertise, denied scan abnormalities	no	yes	Unfounded			
2	2023	Ankle	Orthopedic surgeon	7,5	Incorrect diagnosis and treatment, discrimination based on age, no informed consent	no	yes	Unfounded			
3	2023	Elbow	Orthopedic surgeon	13,1	Not discussing the risks, severing a nerve, not taking patient seriously, incorrect information, incorrect diagnosis, not taking responsibility, the reason the patient can't work	no	yes	Unfounded			
4	2023	Knee	Orthopedic surgeon	9,5	Not informing the patient about a complication, incorrect treatment, not performing additional examinations	no	yes	Unfounded			
5	2022	Shoulder	resident	9,1	Not performing additional examinations, incorrect treatment, no informed consent, insufficient documentation	yes	yes	Unfounded		yes, rejected	Patient
6	2021	Spine	Orthopedic surgeon	8,2	Incorrect documentation	no	yes	Unfounded		yes, rejected	Patient
7	2021	Shoulder	Orthopedic surgeon	10,5	Assessment was not independent and incomplete, facts and diagnoses are mostly based on assumptions and hypotheses, downplaying injuries	yes	yes	Unfounded		yes, rejected	Patient
8	2021	Shoulder	Orthopedic surgeon	7,6	Not performing additional examinations, incorrect interpretation of MRI	no	yes	Unfounded		yes, rejected	Patient
9	2023	Ankle	Orthopedic surgeon	10,3	Not acting in best interest of patient, not thoroughly investigating the problem	no	yes	Unfounded			
10	2023	Ankle	Orthopedic surgeon	10,3	Not addressing the adverse effects following the operation, not communicating the findings to the family physician	no	yes	Unfounded			
11	2023	Ankle	Orthopedic surgeon	10,3	No informed consent, incorrectly performed operation, not providing information	no	yes	Unfounded			
12	2021	Ankle	Orthopedic surgeon	10,6	Withholding information, inadequate documentation, providing incorrect	no	yes	Unfounded		yes, rejected	Patient

					and misleading information, putting own interests above the patient					
13	2021	Knee	Orthopedic surgeon	11,1	Not ordering additional imaging, incorrect diagnosis, not referring, instructing a colleague to ready their judgement	no	yes	Unfounded	yes, rejected	Patient
14	2021	Knee	Orthopedic surgeon	11,1	Incorrect diagnosis, sending a referral letter against the patient's wishes	no	yes	Unfounded	yes, rejected	Patient
15	2021	Knee	Orthopedic surgeon	11,1	Incorrect diagnosis, incorrect information, not listening as he had his judgement ready	no	yes	Unfounded	yes, rejected	Patient
16	2021	Knee	Orthopedic surgeon	11,8	Incorrectly performed operation, incorrect indication, inadequate documentation, marking symptoms as psychiatric, prescribing incorrect medication, delaying the complaint the patient filed to the hospital	no	yes	Unfounded	yes, rejected	Patient
17	2022	Oncology	Orthopedic surgeon	5,8	Not performing a biopsy, not consulting to investigate metastases	yes	yes	Unfounded		
18	2022	Hand	Orthopedic surgeon	8,4	Incorrect method of diagnosis, not ordering additional examinations	no	yes	Unfounded		
19	2022	Hand	Orthopedic surgeon	10,2	Not discussing the risks, selecting a high-risk operation, incorrect operation execution	no	yes	Unfounded		
20	2022	Hip	Orthopedic surgeon	6,5	Incorrect hip placement, complication of wound fluid, too many people in training were present which distracted the surgeon	no	yes	Unfounded		
21	2022	Oncology	Orthopedic surgeon	10	Incorrect diagnosis and documentation, not taking the patient and her family seriously	no	yes	Unfounded		
22	2021	Shoulder	Orthopedic surgeon	6,3	Not referring, not performing additional examinations, insufficient information about the operation, mistreatment	no	yes	Unfounded	yes, rejected	Patient
23	2022	Shoulder	Orthopedic surgeon	7,8	Insufficient examination and documentation, incorrect treatment, no informed consent,	yes	yes	Unfounded		
24	2020	Hip	Orthopedic surgeon	7,6	No informed consent, inexperienced to perform operation, incorrect execution of operation	no	yes	Unfounded	yes, rejected	Patient
25	2022	Shoulder	Orthopedic surgeon	8,6	Not adhering to standards to prevent infection, not taking hemiplegia into account during surgery, inadequate postoperative care and treatment	yes	yes	Unfounded		

26	2021	Hip	Orthopedic surgeon	8,7	No informed consent, limited communication, incorrect diagnosis and treatment	no	yes	Unfounded		
27	2021	Knee	Orthopedic surgeon	12,3	Mistreatment, not substantiating his opinion, not visiting the patient during admission, withheld information	yes	yes	Unfounded		
28	2021	Spine	Orthopedic surgeon	7,8	Incorrect diagnosis, insufficient information on treatment and complications, caused permanent damage	yes	yes	Unfounded		
29	2020	Hip	resident	15,3	Prescribed opiates despite an allergy, did not admit her to the hospital	no	yes	Unfounded		
30	2019	Knee	resident	6,9	Not reporting the abnormal lab values and complications to supervisor, incorrect time-out procedure	no	yes	Unfounded	yes, rejected	Patient
31	2019	Ankle	Orthopedic surgeon	6,4	Insufficient expertise, incorrect information and patient folder, inadequate documentation	no	yes	Unfounded	yes, rejected	Patient
32	2019	Knee	resident	6,4	Insufficient expertise, incorrect information and patient folder, inadequate documentation	no	yes	Unfounded	yes, rejected	Patient
33	2019	Pediatrics	Orthopedic surgeon	10,2	Incorrect diagnosis and execution, insufficient information, inadequate postoperative care	no	yes	Unfounded	yes, rejected	Patient
34	2021	Knee	Orthopedic surgeon	5,3	Incorrect operation indication and prosthesis placement, insufficient information, abandoned the patient	yes	yes	Unfounded		
35	2020	Ankle	Orthopedic surgeon	15	Incorrect operation execution	yes	yes	Unfounded	yes, rejected	Patient
36	2020	Knee	Orthopedic surgeon	12,6	Prolonged waiting times, operated by a different surgeon than discussed, not acting with care	no	yes	Unfounded	yes, rejected	Patient
37	2020	Knee	Orthopedic surgeon	8,9	Abandoning the patient, not taking her seriously, inadequate postoperative care, lack of transparency	no	yes	Unfounded		
38	2020	Spine	Orthopedic surgeon	4,6	Not registered and competent to perform surgery, not providing information	no	no	Unfounded		
39	2019	Shoulder	Orthopedic surgeon	13,1	Ignoring symptoms, not performing a reoperation or additional imaging, conflicting statements to colleagues, not discussing the risks, not saying sorry	yes	yes	Unfounded	yes, rejected	

40	2019	Hip	Orthopedic surgeon	7,4	Incorrect tendon suture during THP, not acknowledging his mistake	no	yes	Unfounded		
41	2019	Knee	Orthopedic surgeon	8,4	Incorrect TKP placement, denying his mistake	yes	yes	Unfounded		
42	2019	Knee	Orthopedic surgeon	8,4	Not documenting that a complication occurred	yes	yes	Unfounded		
43	2018	Knee	Orthopedic surgeon	5,7	Insufficient documentation, not discussing alternatives and complications, acted negligently	no	yes	Unfounded	yes, rejected	Patient
44	2017	Hip	Orthopedic surgeon	2,6	Incorrect operation positioning and handover, insufficient information and documentation, not reporting the complication	yes	yes	Unfounded	yes, rejected	Patient
45	2018	Hand	Orthopedic surgeon	5,5	Ignored radiologist report, incorrect diagnosis, not performing additional examinations, not adhering to the physician oath	no	yes	Unfounded		
46	2018	Spine	Orthopedic surgeon	11,3	Not acknowledging mistakes, brain damage during second surgery, not answering correspondence, not operating	no	yes	Unfounded		
47	2017	Ankle	Orthopedic surgeon	12,6	Incompetent physician performed operation, incorrect instructions, inadequate postoperative care, ignored symptoms, showing false images	no	yes	Unfounded	yes, rejected	Patient
48	2017	Ankle	Orthopedic surgeon	12,6	Incompetent physician performed operation, insufficient information, not discussing treatment options in a timely manner	no	yes	Unfounded	yes, rejected	Patient
49	2018	Oncology	Orthopedic surgeon	5,3	Not prescribing pain medication and oxygen, mistreatment, not answering questions, incorrect discharge	no	yes	Unfounded		
50	2017	Ankle	Orthopedic surgeon	7,5	Not plastering the ankle following arthrodesis, not recognizing infection and performing follow-up	no	yes	Unfounded	yes, rejected	Patient
51	2017	Knee	Orthopedic surgeon	8,2	Not disinfecting the knee during surgery	yes	yes	Unfounded	yes, rejected	Patient
52	2017	Ankle	Orthopedic surgeon	7,5	Not plastering the ankle following arthrodesis, not recognizing infection and performing follow-up	no	yes	Unfounded	yes, rejected	Patient
53	2017	Hand	Orthopedic surgeon	9,6	Insufficient information, surgical error, negligent treatment	yes	yes	Unfounded	yes, rejected	Patient
54	2017	Knee	Orthopedic surgeon	5,1	TKP with nickel despite an allergy	yes	yes	Unfounded	yes, rejected	Patient
55	2018	Other	Orthopedic surgeon	4	No informed consent, disrespectful	yes	yes	Unfounded		

56	2016	Knee	Orthopedic surgeon	6,5	Incorrect documentation and discharge, removing plaster too early, not referring, inadequate follow-up care	yes	yes	Unfounded	yes, rejected	Patient
57	2016	Shoulder	Orthopedic surgeon	6,1	Inadequate postoperative care, delayed response to symptoms	no	yes	Unfounded	yes, rejected	Patient
58	2016	Knee	Orthopedic surgeon	9,9	Not discussing risks, not being able to work after operation	no	yes	Unfounded		
59	2016	Knee	Orthopedic surgeon	9,9	Not discussing risks, not being able to work after operation	no	yes	Unfounded		
60	2016	Knee	Orthopedic surgeon	14,3	Operated without additional imaging, used excessive force intraoperatively to bend the leg	yes	yes	Unfounded		
61	2016	Knee	Orthopedic surgeon	14,3	Not competent to operate, incorrect information, disrupted the medial ligament, operated without additional imaging	yes	yes	Unfounded		
62	2016	Knee	Orthopedic surgeon	14,3	Not competent to operate, no indication for operation	yes	yes	Unfounded		
63	2016	Knee	Orthopedic surgeon	12,8	Operated the wrong side, insufficient communication	yes	yes	Unfounded		
64	2016	Knee	Orthopedic surgeon	8,5	Incorrect diagnosis and treatment	no	yes	Unfounded		
65	2016	Hip	Orthopedic surgeon	8	No informed consent, incorrect examinations and documentation, mistreatment	no	yes	Unfounded		
66	2016	Hip	Orthopedic surgeon	11,6	No complaints against physician	no	yes	Unfounded		
67	2016	Hand	resident	11,8	Incorrect diagnosis and information, not referring,	yes	yes	Unfounded		
68	2015	Hip	Orthopedic surgeon	8,6	Operated without indication	yes	yes	Unfounded	yes, rejected	Patient
69	2015	Hip	Orthopedic surgeon	8,6	Operated without indication	yes	yes	Unfounded	yes, rejected	Patient
70	2014	Knee	Orthopedic surgeon	9,7	Deviating from protocols, not performing additional examinations	yes	yes	Unfounded	yes, rejected	Patient
71	2015	Spine	Orthopedic surgeon	10,5	Not taking diagnosis and symptoms seriously, not treating the patient, not asking a second opinion	no	yes	Unfounded		
72	2015	Ankle	Orthopedic surgeon	7,1	No informed consent, not respecting patient privacy, not consulting patient	yes	yes	Unfounded		
73	2015	Hip	Orthopedic surgeon	15,9	No informed consent, not competent to operate, incorrect operation	yes	yes	Unfounded		
74	2015	Ankle	Orthopedic surgeon	8,9	Not performing additional examinations, incorrect documentation and history and	yes	yes	Unfounded		

					execution of surgery, incorrect diagnosis and medication					
75	2014	Ankle	Orthopedic surgeon	13	No informed consent, limited information, no operation indication, incorrect operation	no	yes	Unfounded	yes, rejected	Patient
76	2014	Shoulder	Orthopedic surgeon	9,7	Insufficient history, misuse of information, not reading recent literature, not referring, inadequate documentation	no	yes	Unfounded		
77	2014	Shoulder	Orthopedic surgeon	9,7	Acted careless and negligent	no	yes	Unfounded		
78	2014	Pediatrics	Orthopedic surgeon	7,9	Incorrect documentation and communication	no	yes	Unfounded		
79	2014	Hip	Orthopedic surgeon	7,4	Not taking symptoms seriously, incorrect treatment, not interested in patient	no	yes	Unfounded		
80	2014	Hand	Orthopedic surgeon	7,3	Incorrect treatment resulting in invalidity	no	yes	Unfounded		
81	2014	Hip	Orthopedic surgeon	16,9	Not considering different treatment, mistreatment, incorrect surgery	no	yes	Unfounded		
82	2014	Hip	Orthopedic surgeon	16,9	Not considering different treatment, mistreatment, incorrect surgery	no	yes	Unfounded		
83	2012	Spine	Orthopedic surgeon	19,5	Performed experimental operation, not discussing complications, not referring, failed to intervene in time	yes	yes	Unfounded	yes, rejected	Patient
84	2012	Other	Orthopedic surgeon	12,6	Incomplete and not an objective examination, misrepresenting medical facts, not substantiating his conclusion	no	yes	Unfounded	yes, rejected	Patient
85	2012	Ankle	Orthopedic surgeon	13,2	No physical examination, no additional examinations, incorrect treatment	no	no	Unfounded	yes, rejected	Patient
86	2011	Spine	Orthopedic surgeon	6,2	Incorrect diagnosis, no additional examinations	no	no	Unfounded	yes, rejected	Patient
87	2013	Hand	Orthopedic surgeon	12,6	Incorrect surgical technique, not discussing complications, not listening to patient symptoms	yes	no	Unfounded		
88	2013	Other	Orthopedic surgeon	13	Insufficient additional examinations, short physical exam, not reading notes, incorrect conclusions	no	no	Unfounded		
89	2012	Shoulder	Orthopedic surgeon	9	Not consulting a shoulder expert, not acknowledging his mistake	no	yes	Unfounded	yes, rejected	Patient
90	2012	Hip	Orthopedic surgeon	11,9	Insufficient treatment, not consulting colleague, not checking medication list, not checking on the work of the resident	no	yes	Unfounded	yes, rejected	Patient

91	2013	Other	Orthopedic surgeon	17,4	Improper treatment, not calling the emergency services	yes	yes	Unfounded		
92	2013	Spine	Orthopedic surgeon	7,6	Incorrect diagnosis, insufficient communication, discharged without information	yes	yes	Unfounded		
93	2012	Spine	Orthopedic surgeon	15,2	Not taking patient seriously, forcing to undergo a scan	no	yes	Unfounded	yes, rejected	Patient
94	2013	Other	Orthopedic surgeon	12,7	Incorrect diagnosis	no	yes	Unfounded		
95	2012	Knee	Orthopedic surgeon	12,3	Not performing additional examinations, incorrect treatment, not competent to refer	yes	yes	Unfounded		
96	2012	Ankle	Orthopedic surgeon	11,5	Incorrect operation, inadequate follow-up care	no	yes	Unfounded		
97	2012	Other	Orthopedic surgeon	3	Encouraging suicidal thoughts	no	yes	Unfounded		
98	2012	Hip	Orthopedic surgeon	14,3	Incorrect diagnosis, failed his duty of care, mistreatment, incorrect documentation	yes	yes	Unfounded		
99	2012	Ankle	Orthopedic surgeon	11,5	Incorrect execution of the operation	no	yes	Unfounded		
100	2010	Other	Orthopedic surgeon	9,5	Incorrect diagnosis	no	no	Unfounded	yes, rejected	Patient
101	2012	Hip	Orthopedic surgeon	11,3	Insufficient communication and additional examinations, not consulting colleague, acted too passively, ignored her views	no	yes	Unfounded		
102	2010	Elbow	Orthopedic surgeon	3,3	Operation delay, incorrect incision and suture, insufficient postoperative care	no	no	Unfounded	yes, rejected	Patient
103	2009	Other	Orthopedic surgeon	18,9	Incorrect conclusion based on limited information	no	yes	Unfounded	yes, rejected	Patient
104	2010	Knee	Orthopedic surgeon	10,1	Provided insufficient care	no	yes	Unfounded	yes, rejected	Patient
105	2011	Shoulder	Orthopedic surgeon	10,6	Incorrect diagnosis, incorrect operation, contradictory information, no additional examinations	yes	yes	Unfounded		
106	2011	Hip	Orthopedic surgeon	10,8	Insufficient examinations, documentation and information, not taking medical history into account for surgery, no informed consent, mistreatment,	no	yes	Unfounded		
107	2011	Ankle	Orthopedic surgeon	9,6	Longer operation, too many students present, insufficient postoperative care, mistreated	no	yes	Unfounded		
108	2010	Knee	Orthopedic surgeon	10	Incorrect treatment, discharge, documentation, sexual intimidation	no	yes	Unfounded	yes, rejected	Patient

109	2009	Ankle	Orthopedic surgeon	11,8	Caused neurological damage, did not inform about the complication, mistreatment	no	yes	Unfounded		
110	2010	Knee	Orthopedic surgeon	11,1	Mistreatment	no	yes	Unfounded		
111	2009	Hip	Orthopedic surgeon	8,3	Incorrect operation	no	yes	Unfounded	yes, rejected	Patient
112	2009	Ankle	Orthopedic surgeon	8	Insufficient information, causing mobility impairment	no	yes	Unfounded	yes, rejected	Patient
113	2009	Other	resident	15,4	Providing insufficient care and communication	yes	yes	Unfounded	yes, rejected	Patient
114	2010	Knee	Orthopedic surgeon	24,2	Incorrect surgery method, not acknowledging mistakes	yes	yes			

Supplementary Table S2. A summary of all founded verdicts

Case	Year	Subspecialty	Defendant	Time between filing and verdict (months)	Allegation	Attorney patient	Attorney defendant	Verdict	Measure	Appeal	Appellant
1	2021	Spine	Orthopedic surgeon	6,8	Removing too much bone and ignoring cord compression, inadequate documentation, not present at consultation with colleagues not tapering medication before discharge	yes	yes	Partially Founded	Warning		
2	2021	Spine	Orthopedic surgeon	6,8	Operated incorrect side, delayed additional examinations, removing too much bone and ignoring cord compression, inadequate documentation	yes	yes	Partially Founded	Warning		
3	2018	Shoulder	Orthopedic surgeon	9,6	Incorrectly performed 3 operations, ignored pain symptoms and gave insufficient information, made contradicting statements	yes	yes	Partially Founded	Warning	yes, warning unchanged	orthopedic surgeon
4	2020	Hip	Orthopedic surgeon	4,4	THP performed with limited information and incorrect diagnosis, not adhering to guidelines, not discussing the risks	yes	yes	Partially Founded	Reprimand		
5	2018	Hip	Orthopedic surgeon	5,9	Insufficient communication and information, going beyond his area of expertise, derogatory remarks	yes	yes	Partially Founded	Reprimand	yes, reprimand changed to warning	orthopedic surgeon
6	2018	Hip	Orthopedic surgeon	5,9	Insufficient communication and information, going beyond his area of expertise, derogatory remarks	yes	yes	Partially Founded	Warning	yes, rejected	orthopedic surgeon
7	2017	Spine	Orthopedic surgeon	18,3	Incorrect history and operation indication, controversial surgical technique against guidelines, not referring to a colleague before his absence	yes	yes	Founded	Reprimand	yes, reversed reprimand	orthopedic surgeon
8	2018	Other	resident	3,9	Accessing electronic health record without a doctor patient relationship, sharing this information	yes	yes	Partially Founded	Warning		
9	2017	Spine	Orthopedic surgeon	10,1	Extended operation time, cauda equina syndrome during surgery, insufficient postoperative care	yes	yes	Partially Founded	Suspension of 6 months	yes, suspension unchanged	orthopedic surgeon
10	2017	Spine	Orthopedic surgeon	7,7	Insufficient information and documentation, no informed consent, intraoperative paraplegia, not reporting the complication, perjury statements	yes	yes	Partially Founded	Warning	yes, rejected	patient
11	2017	Ankle	Orthopedic surgeon	9,9	Not initiating contact after causing an intraoperative skin burn, blaming the manufacturer	yes	yes	Partially Founded	Warning	yes, rejected	patient
12	2017	Ankle	Orthopedic surgeon	9,9	Not initiating contact after causing an intraoperative skin burn, blaming the manufacturer	yes	yes	Partially Founded	Warning	yes, rejected	patient
13	2017	Hip	Orthopedic surgeon	7,6	Incorrect positioning intraoperatively and handover, insufficient documentation, not reporting the complication	yes	yes	Partially Founded	Warning		
14	2017	Hip	Orthopedic surgeon	12,2	Insufficient communication and postoperative care, inhumane and uninterested treatment	no	yes	Partially Founded	Warning		
15	2016	Shoulder	Orthopedic surgeon	4,6	Performing a different operation than documented, ignoring echo results, insufficient documentation, destroying patient confidence in medicine	no	yes	Partially Founded	Reprimand		
16	2016	Other	Orthopedic surgeon	12,7	Insufficient documentation	yes	yes	Partially Founded	Warning	yes, warning unchanged	both

17	2016	Spine	Orthopedic surgeon	11,5	Not discussing the operation risks, no informed consent, not performing imaging after placement of pedicle screws in spinal canal	yes	yes	Unfounded		yes, reprimand	patient
18	2016	Hip	Orthopedic surgeon	14	Incorrect diagnosis, not referring, insufficient information and documentation, forgery, incorrect postoperative counseling	no	yes	Partially Founded	Warning		
19	2016	Knee	Orthopedic surgeon	7,2	Not adhering to postoperative guidelines	yes	yes	Founded	Reprimand	yes, reprimand unchanged	orthopedic surgeon
20	2015	Shoulder	Orthopedic surgeon	16	Gross negligence and incompetence during surgery, poor communication, mediating by an incompetent and biased mediator	yes	yes	Partially Founded	Warning		
21	2015	Hip	Orthopedic surgeon	12,2	Incorrect diagnosis, inadequate follow-up care and documentation	yes	yes	Founded	Reprimand		
22	2013	Shoulder	Orthopedic surgeon	17,7	Responding inappropriately and behaving unprofessionally, ignoring findings and opinions from physical therapists	yes	yes	Founded	Warning	yes, warning dismissed	Orthopedic surgeon
23	2013	Shoulder	Orthopedic surgeon	5,3	Incorrect documentation	yes	yes	Unfounded		yes, Warning	Patient
24	2013	Other	Orthopedic surgeon	7,8	Not adhering to professional standard in the professional code for medical consultants, included hurtful and disrespectful passages about the patient	yes	yes	Unfounded		yes, Warning	Patient
25	2014	Ankle	Orthopedic surgeon	8,4	Incorrectly performed hallux valgus operation	yes	yes	Partially Founded	Warning		
26	2014	Spine	Orthopedic surgeon	14	Operated incorrect level, used an outdated technique, provided insufficient information, incorrect treatment, incurring unnecessary costs	no	yes	Partially Founded	Warning		
27	2014	Shoulder	Orthopedic surgeon	8,1	Not adhering to guidelines, ignored patient symptoms causing a necrotic thumb, not consulting surgery, not informing patient and insufficient documentation	yes	yes	Founded	Reprimand		
28	2014	Shoulder	Orthopedic surgeon	7,6	Incorrect documentation and referral, not initiating contact, not informing about the risks	yes	yes	Partially Founded	Warning		
29	2013	Ankle	Orthopedic surgeon	9,4	Plastering an open wound, no informed consent, not ordering additional examinations, causing necrosis for which an amputation was needed, insufficient documentation	yes	yes	Partially Founded	Warning	yes, unchanged	Patient
30	2012	Knee	Orthopedic surgeon	11,5	Insufficient postoperative care after damaging a leg artery, delayed imaging, permanent sensory loss and limited range of motion	no	yes	Founded	Warning	yes, rejected	Orthopedic surgeon
31	2012	Hip	Orthopedic surgeon	13,1	Inadequate postoperative care regarding an abscess after THP, patient died as a result	no	yes	Partially Founded	Reprimand	yes, changed to warning	Both
32	2013	Spine	Orthopedic surgeon	15,9	Operated incorrect level, not discussing the risks, inadequate follow-up care	no	yes	Partially Founded	Warning		
33	2013	Ankle	Orthopedic surgeon	11	Not discussing the risks, errors during surgery, not taking patient seriously	yes	yes	Partially Founded	Warning		
34	2013	Hip	Orthopedic surgeon	4,9	Not discussing the risks, using different technique, severed nerves during surgery, financial consequences due to impaired mobility	yes	yes	Partially Founded	Warning		
35	2012	Knee	Orthopedic surgeon	12,5	Not discussing the risks, incorrect operation execution	yes	yes	Founded	Warning		

36	2012	Spine	Orthopedic surgeon	6,3	Incorrect operation execution, inadequate communication, mistreatment	yes	yes	Partially Founded	Suspension for 1 month		
37	2012	Hip	Orthopedic surgeon	10,3	Not discussing the surgery, no informed consent, incorrectly continued the surgery, not reporting the complication, insufficient documentation	yes	yes	Partially Founded	Warning		
38	2010	Spine	Orthopedic surgeon	13,7	Incorrect operation, ignoring findings from colleagues, no informed consent, inadequate documentation and communication	yes	yes	Partially Founded	Reprimand	yes, reprimand unchanged	Both
39	2010	Spine	Orthopedic surgeon	13,8	Incorrect operation, ignoring findings from colleagues, no informed consent, inadequate documentation and communication	yes	yes	Partially Founded	Reprimand	yes, reprimand unchanged	Orthopedic surgeon
40	2011	Other	Orthopedic surgeon	11,3	Ignoring guidelines	yes	yes	Founded	Warning		
41	2011	Spine	Orthopedic surgeon	9,8	Not communicating critical findings, not consulting colleagues, incorrect documentation, damaging nerves, not skilled to operate, concealing and not recognizing the complication	yes	no	Partially Founded	Suspension of 1 year		
42	2009	Hip	Orthopedic surgeon	14,2	Not acknowledging the VTE	yes	yes	Partially Founded	Warning	yes, rejected	Patient
43	2009	Hip	Orthopedic surgeon	9,8	Careless action and late intervention significantly affecting life quality, not referring, not attempting to regain trust	no	yes	Partially Founded	Warning		
44	2010	Knee	Orthopedic surgeon	16,7	Incorrect operation, ignoring patient's preference	no	no	Founded	Warning		
